# Biodiesel production through transesterification of waste Pistacia- Terebinthus Oil by pharmaceutical waste as a heterogeneous catalyst: A sustainable solution for reducing external costs

**DOI:** 10.1016/j.heliyon.2024.e34404

**Published:** 2024-07-17

**Authors:** Saman Rashidi, Ramin Tahmasebi-Boldaji, Aref Ahmadian Baghbadarani, Majid Baghdadi, Omid Tavakoli, Abdolreza Karbassi, Akram Avami

**Affiliations:** a Department of Environmental Engineering, Graduate Faculty of Environment, University of Tehran, Tehran, Iran; b Department of Chemical Engineering, College of Engineering, University of Isfahan, P.O. Box 81746-73441, Isfahan, Iran; c School of Chemical Engineering, College of Engineering, University of Tehran, Tehran, 14176, Iran; d Department of Energy Engineering, Sharif University of Technology, Azadi Ave, Tehran, 14565-114, Iran

**Keywords:** Biofuel, Pistacia-terebinthus (PT), Heterogeneous catalyst, Pharmaceutical waste, External costs

## Abstract

A catalyst from the pharmaceutical waste of calcium and magnesium tablets was synthesized for biodiesel production from waste Pistacia-Terebinthus (PT) oil with the aim of creating added value and presenting a new approach for the management of such wastes. For this purpose, magnesium and calcium tablet wastes with a mass ratio of 70:30 (wt%) were calcined. The catalyst was investigated by several methods, such as thermal gravimetric analysis, X-ray diffraction, Fourier transform infrared spectroscopy, field emission scanning electron microscopy, Brunauer-Emmett-Teller analysis, X-ray photoelectron spectroscopy, and CHNS/O elemental analysis. The high specific surface area of the catalyst confirms that the utilized synthesis method resulted in the formation of a high number of active sites in its structure, which allows it to function as a suitable catalyst for this reaction. Furthermore, the impact of effective parameters on the treansestrification reaction was optimized and investigated by designing the experiments and applying the RSM method. The maximum mass yield of 96 % was obtained in optimal conditions (temperature of 70 °C, catalyst loading of 4.498 wt%, methanol:oil ratio of 1.968 (vol:vol), and reaction time of 120 min). The reusability of the catalyst was investigated in four successive cycles. The mass yield of the last test declined from 96 % to 71.4 %. Gas chromatography-mass spectrometry analysis of the produced biofuel revealed that it comprises 91.37 % methyl ester compounds (64.28 % 12-Octadecenoic Acid, Methyl Ester). To evaluate the external costs of biofuel (B100) and compare it with diesel, combustion simulation was done with Diesel-RK software, which showed that its external costs were 0.05388 (€/Lit fuel) less than those of diesel.

## 1 Introduction

Population growth and the industrialization of the world have led to an increase in demand and an increase in the consumption of fossil fuels. Since the consumption of fossil fuels destroys the environment, researchers have been led to find renewable and environmentally friendly sources [1]. The consumption of fossil fuels will increase greenhouse gases such as carbon dioxide, which leads to the challenges of climate change; therefore, replacing these energy sources with renewable sources is inevitable [2,3].

Because of their physical and chemical properties, vegetable oils resemble diesel fuel, which is an alternative fuel that engines can use for combustion [4,5]. Due to the high viscosity of vegetable oil, there are various processes such as dilution, transesterification, emulsification, and pyrolysis to improve it, and the transesterification reaction is the most efficient method that can be used without changing its product in diesel engines [6,7]. The transesterification product of vegetable oils with methanol is known as biodiesel, which contains fatty acid methyl ester (FAME) [8]. The transesterification reaction is affected by several important parameters, such as reaction temperature, methanol-to-oil ratio, reaction time, and amount of catalyst [9].

Various oils can be used as raw materials in the transesterification process, both oils that are included in the human diet (such as sunflower, canola, and soybean oils) and non-edible oils such as eucalyptus oil, waste cooking waste, and pistacia-terebinthus oil. Efforts should be focused on the use of non-edible oils to have the least conflict with the human food basket. Pistachio-terbintus oil is obtained from the fruit of the pistachio-terbintus tree. This tree has globular fruits that are green in color, turn purplish-green when ripe, and have aromatic gum. This tree grows in the city of Ilam in Iran (the South Zagros mountain area) and parts of Asia. The Iran Agriculture Ministry claims that this tree is one of the wild species, having a range of about 2 million hectares and an estimated population of 80 million. This tree yields 25 kg of fruit on average every year. Pistacia-Terebinthus fruit oil makes up about 35 % of the total. Its gum, fruit, and oil have been used in medicine for a long time, but little attention has been paid to it for biofuel production. The used oil waste was collected from the local oil production factory located in Ilam City, which was oil production line waste.

Transesterification is done with homogeneous and heterogeneous catalysts. The simplest extraction method requires a homogeneous catalyst, but with respect to the high production costs that are caused by more product washing and laborious separation of the catalyst, heterogeneous catalysts are preferred [10]. Calcium oxide is one of the most suitable options among heterogeneous catalysts because it is cheap, has a higher basicity, and is less corrosive than homogeneous catalysts [11]. CaO sources can be different, such as animal bones and egg shells [12]. For example, for the preparation of CaO catalyst, the fish head bones (croaker fish) and waste shells of sea snails have been used as catalysts [4,5]. In addition to these, the catalytic MgO has the advantage of being able to increase the efficiency of the transesterification reaction to a very favorable level [12].

The United States generates more than 3.5 million tons of medical waste [13]. Also, in Iran, according to the report of the Food and Drug Organization, Iran's capital, Tehran province, had more than 3000 tons and 80,000 L of pharmaceutical-chemical waste as solids and liquids, respectively, in 2022. The use of these drugs is mostly in hospitals and also at home with a doctor's prescription. Pharmaceutical industries and hospitals generate the majority of this waste. The mentioned statistics are only for chemical-pharmaceutical waste produced from hospitals; pharmaceutical companies, as well as the amount of waste produced at home, are not included in these statistics. These wastes must be disposed of according to the regulations of the Ministry of Health in Iran. The disposal methods used in Iran are mostly incineration and landfills, which cause pollution such as air, soil, water, and landscape pollution. The proposed method can be a new alternative to the current waste management approach. Also, instead of using this method, it can be shown that it is possible to create added value through the production of a valuable product, such as a catalyst. Therefore, the use of pharmaceutical waste (such as calcium and magnesium tablets or zinc epoxide ointment, etc.) that can be converted into catalysts is an attractive choice for catalyst production.

The aim of this study is to present a new method for pharmaceutical waste management, creating added value through the synthesis of catalyst from the waste of magnesium and calcium pharmaceutical tablets. Therefore, the catalyst was produced from this waste by the calcination method. Oil waste from the production line of the PT oil factory as feed, methanol as solvent, and the prepared catalyst were used for the production of biofuel by the transesterification method. The use of a higher amount of MgO in the synthesized catalyst was chosen due to its impact on the efficiency of biodiesel production. Then the catalyst was examined in different ways in terms of chemical and physical properties. In addition, by using Design-Expert software and response surface methodology (RSM), the effect of important parameters such as temperature, catalyst loading, methanol:oil ratio, and reaction time on biodiesel mass yield was studied. The product was analyzed in terms of chemical content. Combustion simulation was done in a six-cylinder engine with a 1500 rpm nominal engine speed using Diesel-RK software. Environmental emission factors were investigated, and the external cost of the produced biodiesel was calculated and compared with diesel.

## 2 Methodology

### 2.1 Materials

The magnesium oxide (MgO) and calcium carbonate (CaCO3) waste pills were collected from pharmacies in Iran's capital, Tehran. In addition, high-purity methanol was acquired from Merck in Germany. The Pistacia-Terebinthus (PT) waste oil was collected from oil factories in Ilam (a region in western Iran) and used for transesterification experiments and biofuel production.

### 2.2 Catalyst preparation

First of all, the waste of calcium carbonate and magnesium oxide tablets was sieved to 250 μm. In the second stage, MgO and CaCO_3_ were mixed with a mass ratio of 70:30 (wt%) and stirred for 2 h in deionized water at room temperature to obtain a suspension. According to the previous article, it was concluded that the performance and yield of MgO is higher than CaO and it was proved by the GC-MS test [6].

After that, it was placed in an oven at a temperature of 120 °C for 24 h to evaporate all its moisture. In the end, CaCO_3_ and MgO were calcined at 800 °C for 3 h in a furnace. Calcination is effective in converting calcium carbonate to calcium oxide, removing additional ingredients from magnesium oxide tablets, and increasing specific surface area and porosity to achieve the desired catalyst.

### 2.3 Catalyst characterization

A TGA (STA6000 device) examination was conducted from 25 to 900 °C to examine the effects of heat treatment on the catalyst's characteristics. FT-IR (Tensor II device) at a wavelength between 400 and 4000 cm^−1^ was used to identify catalyst bonds. FESEM (model MIRA3 made by the TESCAN company) was used to investigate surface morphology. BET (BELSORP Mini II, made by BEL Company Japan) was used to investigate specific surface areas. X-ray diffraction (D8ADVANCE XRD device made by Bruker, Germany) was used to identify compounds and phases. XPS Thermofisher ScientifiC K-ALPHA spectra were deconvoluted using CasaXPS software based on the Gaussian-Lorentzian composite function after Shirley background subtraction. All of the peaks were calibrated with respect to the standard C1s binding energy peak of 285.5 eV. Furthermore, ICP-MS (Agilent 7500) was used to calculate the amounts of leached calcium and magnesium.

### 2.4 Transesterification method

To eliminate particle contaminants, the waste PT oil was filtered. It was also heated to 110 °C to eradicate any potential moisture. The transesterification reaction was carried out in a flask that was equipped with a condenser, thermometer, and magnetic stirrer. In order to increase the acceleration of the transesterification reaction, the flask containing reactants and catalyst was placed in a water bath. The effects of important parameters, including catalyst loading, temperature, reaction time, and methanol:oil ratio, were investigated. The catalyst was distributed in methanol at 40 °C and agitated with a magnetic stirrer at 500 rpm to perform the transestrification method. The catalyst and methanol were then mixed with PT oil, and experiments were carried out in line with Table 3.

After the end of the reaction, the contents of the flask were poured into a decanter, and the three phases of catalyst (lower phase), glycerol (middle phase), and biofuel (upper phase) were separated from each other. Then the desired product was centrifuged to completely remove the heterogeneous catalyst. To remove methanol and residual moisture, the resulting biofuel was washed three times with methanol and dried in an oven at 110 °C.

Eventually, the produced methyl esters were analyzed by GC-MS (Instrument Specifications: Manufacturer Company: Agilent Technologies, GC System: 7890A, USA) and a CHNS analysis using a 2400 series device ІІ CHNS/O elemental analysis made in the USA was used to determine the elements. A vibrating viscometer (SVM 3000/G2) and a densimeter (DMA 4500) were used to measure the viscosity and density of the biofuel, respectively.

The mass yield of biofuel was calculated using Eq. (1) [7]. In the last step, the input parameters were optimized.

(1) MassYield%=(Actualweightofbiofuel/Weightofoilused)×100

### 2.5 Experimental design

The matrix test was designed using the response surface methodology. First of all, the variables were selected according to the literature review. Then, the effect of the factors on the output or response of the process and the adequacy of the model were evaluated using ANOVA analysis, which is an important statistical technique. This analysis can classify factors and inputs based on importance and identify the parameters that have the greatest impact on the output [8]. In the third step, a set of data was programmed based on a design (Central Composite Design (CCD)). Finally, a mathematical equation based on the Taylor series, the expanded second-order polynomial model, was used, which is as follows (Eq. (2)) [9]:

(2) Y=β0+∑βiXi+∑βiiXi2+∑βijXiX+je

In the second-order polynomial model, Y, X_i,_ and X_j,_ β_i,_ β_ii,_ β_ij,_ β_0,_ and e are the response value, independent variables, linear coefficient, quadratic coefficient, interaction coefficient, constant, and the error term, respectively. The coefficients of the equation are obtained, and the system of equations is solved using the least squares method. Also, in the continuation of the design process, the input parameters are optimized. Then, the prediction of the response is done by solving the equation, and the compatibility of the model is determined with the experimental data. To evaluate the significance of the model and its quantitative prediction ability, Fisher's test (F-value) and the R^2^ coefficient were used, respectively. In this study, the CCD method with four input factors was used. The input factors and their levels are given in Table 1.

**Table 1:** Actual and coded levels of the independent variables.

		Levels	
Independent variables	−1	0	+1
A-Temperature (°C)	40	55	70
B-Catalyst loading (wt%)	1	4	7
C-Methanol: Oil	0.5	1.75	3
D- Reaction Time	45	82.5	120

## 3 Results and discussion

### 3.1 Characterization of the catalyst

#### 3.1.1 Thermal gravimetric analysis (TGA)

**Fig. 1:**
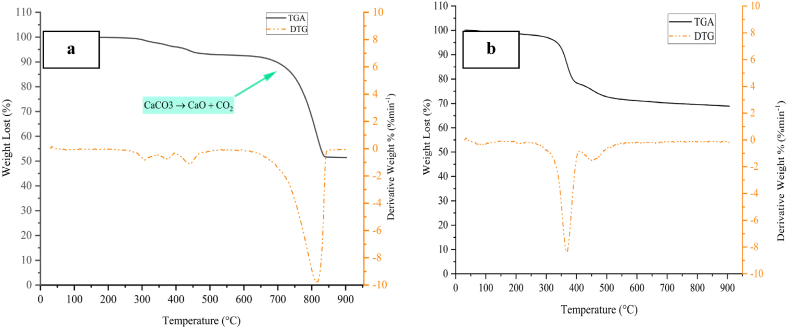
Thermogram for CaCO_3_ (a) and MgO (b).

Fig. 1 (a, b) depicts TGA and DTG curves for CaCO_3_ and MgO. Diagram (a) indicates that decomposition has taken place after 600 °C. The initial weight loss can be due to the removal of possible moisture in it at temperatures below 150 °C that Other studies confirm this result [10,11].The main stage of weight loss at a temperature of 600–850 °C is related to the decomposition of CaCO_3_ and conversion to CaO, which DTG confirms it with an endothermic reaction [12]. The maximum weight loss in calcium tablets is about 48 %. The main stage of weight loss at a temperature of 600–850 °C is related to the decomposition of CaCO_3_ and conversion to CaO, which DTG confirms with an endothermic reaction [12]. The maximum weight loss in CaO is about 48 %. The analysis of Diagram (b) indicates that the moisture and impurities in the sample are removed up to 200 °C in the first step; barros et al. and mendonca et al. also have same result [13,14]. The main step of weight loss, which is confirmed by the DTG diagram, is in the range of 250–450 °C, which is caused by the removal of organic compounds (microcrystalline cellulose and stearic acid). The investigation shows that the sample lost a maximum of 31 % of its weight and then reached stability.

#### 3.1.2 X-ray diffraction (XRD)

**Fig. 2:**
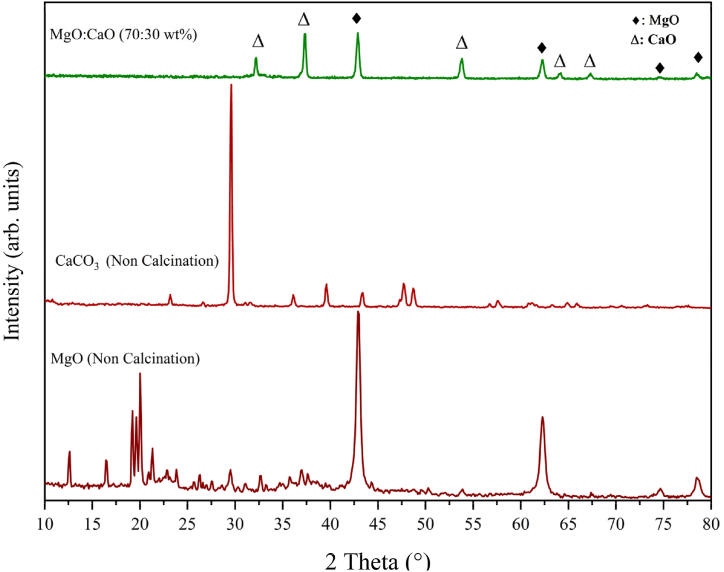
XRD analysis of non-calcinated MgO and CaCO_3_ and MgO:CaO (70:30 wt%) calcined catalyst.

The X-ray diffraction pattern (XRD) of CaCO_3_ and MgO waste tablets prior to and subsequent to calcination is shown in Fig. 2. Calcination successfully converts impure CaCO_3_ and MgO to pure polycrystalline CaO and MgO. The numerous sharp and narrow peaks in the image are indicative of the polycrystalline structure and the activity of the catalyst [15,16]. For the calcined catalyst, the peaks at 2θ = 32.2°, 37.4°, 53.8°, 64.2° and 67.3° and the peaks at 2θ = 36.8°, 42.9°, 62.3°, 74.5° and 78.5° are related to the CaO and MgO, respectively [17,18]. There is a strong association observed between the standard values of MgO, which correlates to JCPDS card number 87–0653, and the lattice planes (111), (200), (220), (311), and (222) and the standard values of CaO, which correspond to JCPDS card number 37–1497, and the lattice planes (110), (200), (220), (311), (222), and ((400). These CaO peaks are also confirmed by a study that employed a CaO-based catalyst that was taken from a silver croaker [4]. Furthermore, a different investigation using a MgO-based catalyst extracted from limestone confirms the presence of MgO peaks [19]. The crystal structure of MgO and CaO indicates that their crystal system is FCC (Face-Centered Cubic). According to the Scherrer equation, the average crystallite sizes are 120.02 nm and 25.32 nm for the MgO and CaO respectively.

#### 3.1.3 Fourier transform infrared spectroscopy (FT-IR)

**Fig. 3:**
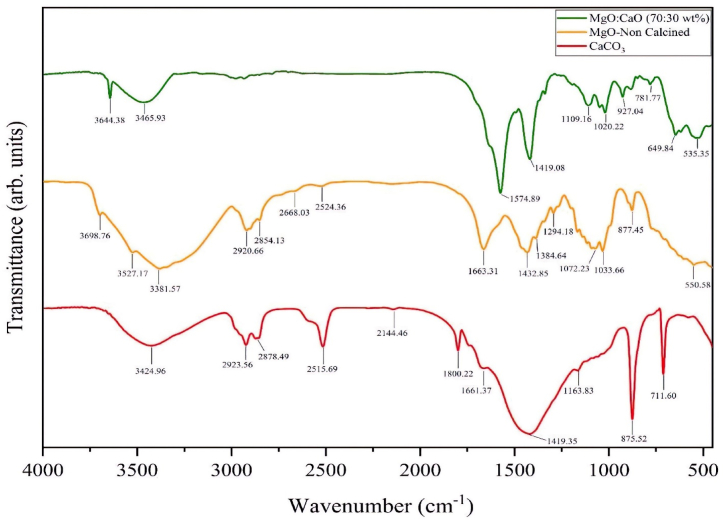
FT-IR analysis of non-calcinated MgO and CaCO_3_ and MgO:CaO (70:30 wt%) calcined catalyst.

The spectra of the catalysts before and after calcination are shown in Fig. 3. A metal-oxygen bending vibration can exist at wavelengths between 433 and 769 cm^−1^ [20,21]. In addition, the wavelength range of cm^−1^ exhibits a stretching Mg–*O*–Mg vibration mode. The wavelengths 3300–3600 cm^−1^ imply a MgO structure, while the wavelength 1431.89 cm^−1^ corresponds to the surface hydroxyl group's bending vibration [22]. Also, the peak between 677 and 487 cm^−1^ and 473 cm^−1^ is related to the Mg–O bond [23,24]. The C–O bond is illustrated by the wavelength of 1419.35 cm^−1^, which establishes the link between the calcium atom and the oxygen-carbonate atom. In addition, the C–O bond can also exist at wavelengths of 711.604 and 875.524 cm^−1^ [25]. On the other hand, the wavelengths 803.206, 1402, and 1085.73 cm^−1^ present peaks that are related to calcium oxide particles, which are attributed to C–O bonds [25]. The peak at the wavelength of 3641.91 cm^−1^ also shows the O–H bond of water adsorbed on the particles' surfaces [25]. According to previous studies, the peaks obtained at the wavelengths shown in Fig. 3 for the MgO:CaO catalyst indicate the formation of MgO and CaO structures.

#### 3.1.4 Field emission scanning electron microscopy (FESEM)

**Fig. 4:**
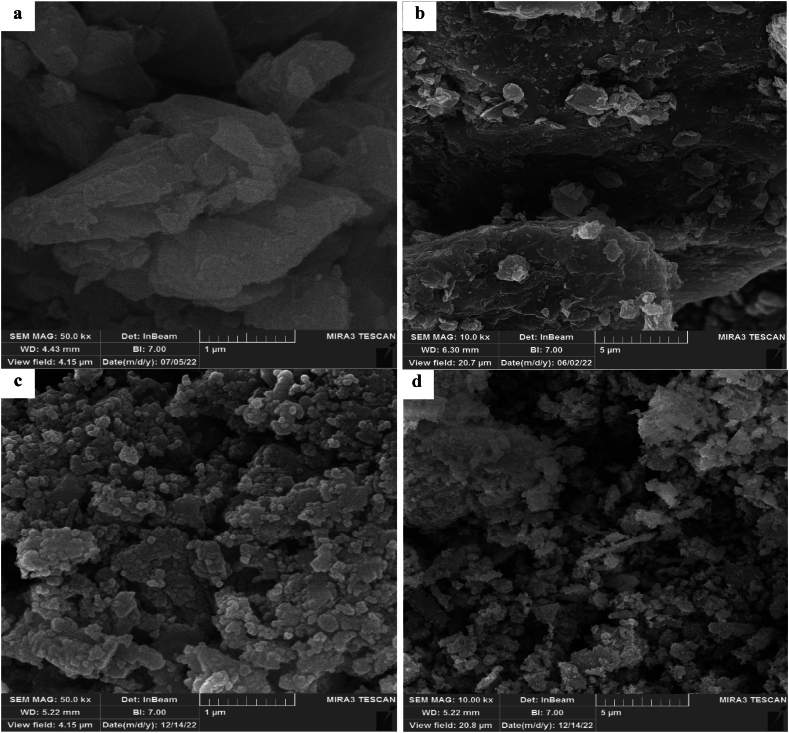
FESEM images of catalyst. (a) non-calcinated CaCO_3_, (b) non-calcinated MgO, (c) and (d) catalyst.

Fig. 4 shows the FESEM images of the catalyst before and after calcination. The images illustrate the morphology of the catalyst surface before and after preparation. Fig. 4 (a, b) shows the catalyst surface before the calcination and Fig. 4 (c, d) after the calcination. Fig. 4 (a, b) clearly illustrates that the particles have a larger size and their specific surface area is smaller. The desired catalyst surfaces after calcination are shown in Fig. 4 (c, d). Morphological properties have changed, and particles with smaller agglomerate sizes have appeared [26].

#### 3.1.5 Brunauer-Emmett-Teller (BET)

**Fig. 5:**
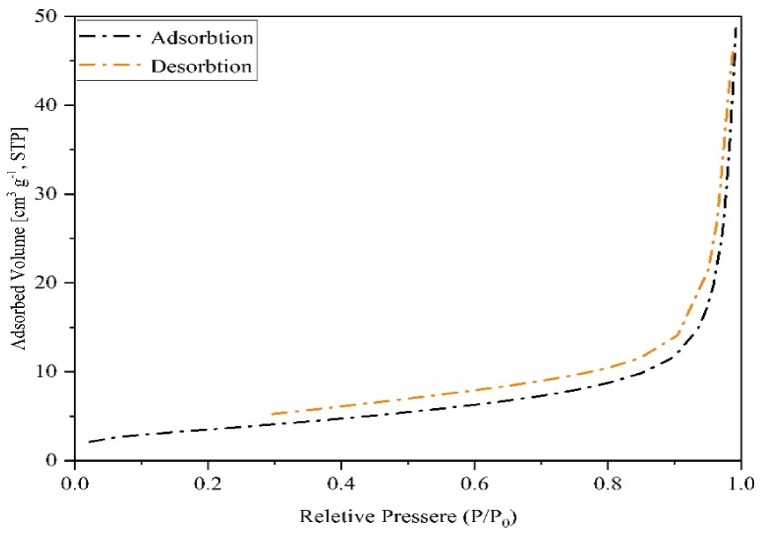
Adsorption-desorption diagram of the catalyst.

Table 4 contains the results of the BET. Fig. 5 shows the adsorption-desorption isotherms of the catalyst surface with N_2_ gas (Table 2). Due to the isotherm obtained using the IUPAC classification and C = 88.06, it was found to be type IV. The capillarity phenomenon, which is related to the H3 type and confirms the presence of mesopores, has resulted in a hysteresis ring [20]. Solids with layered morphology and the distribution of mesopores in the middle of the isotherm indicate the presence of H3-type hysteresis in the solid structure [21,22].

**Table 2:** N_2_ gas physical adsorption of catalysts and tablets.

Sample	Specific surface before calcination (m^2^g^−1^)	Total pore volume before calcination (cm^3^g^−1^)	Total pore volume after calcination (cm^3^g^−1^)	Specific surface after calcination (m^2^g^−1^)
Calcium tablets	1.03	0.02	0.011	3.34
Magnesium tablets	7.77	0.07	0.062	9.35
Catalyst	–	–	0.07	12.72

#### 3.1.6 X-ray photoelectron spectroscopy (XPS)

**Fig. 6:**
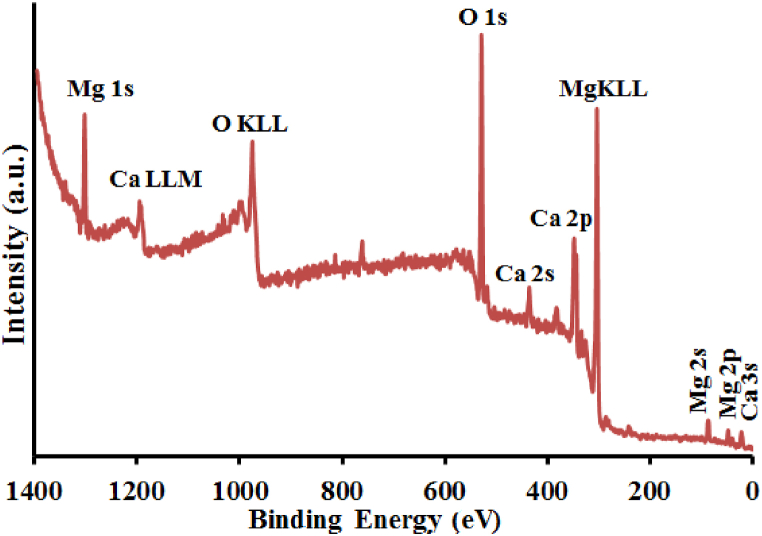
Sample xps spectra.

In the spectrum XPS full scan (survey) of the examined sample in Fig. 6, three specified peaks are observed at bond energies of 533.35 eV, 352.7 eV, and 1032.95 eV, which respectively indicate the presence of O 1s, Ca 2p, and Mg 1s in this compound [27]. The area beneath the relevant peaks in the XPS spectrum was used to estimate the ratio of the sample's constituent elements. The relevant percents for calcium, magnesium, and oxygen are 11.8, 13.6, and 76.4 percent, respectively.

**Fig. 7:**
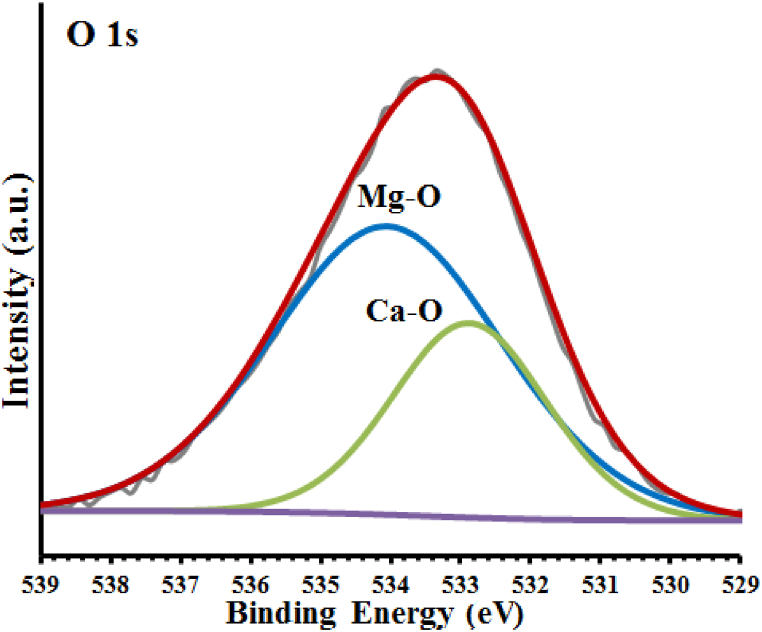
O 1s High-resolution spectra.

Fig. 7 depicts the spectrum of O 1s (XPS high resolution), which has been deconvoluted into two sub-peaks. The peak at 532.88 eV in this sample corresponds to the CaO species (31.02 %), whereas the peak at 534.05 eV belongs to the MgO species (68.98 %) [24].

**Fig. 8:**
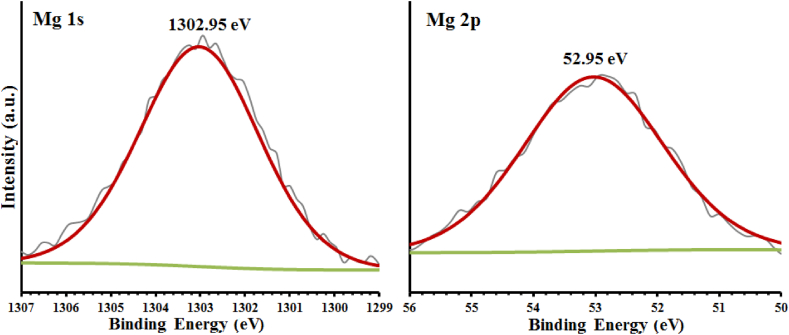
Mg 2p and Mg 1s high resolution spectras.

The spectra of Mg 2p and Mg 1s XPS at high resolution are shown in Fig. 8, which indicates the presence of MgO in this sample [25,28].

**Fig. 9:**
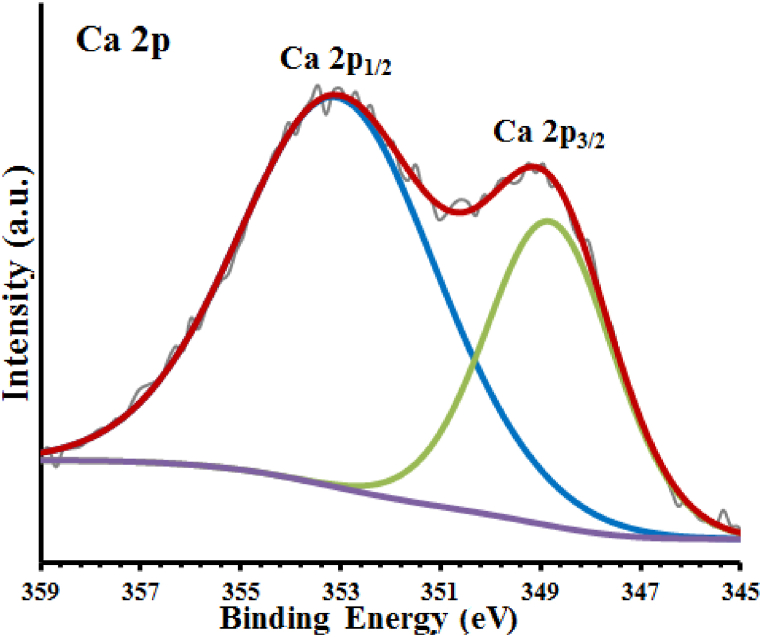
Ca 2p High resolution spectra.

The spectrum of Ca 2p (XPS high resolution) in Fig. 9 shows two specified peaks of Ca 2p3/2 and Ca 2p1/2 at 348.82 eV and 352.1 eV, respectively, and confirms the presence of CaO species in this sample [29].

### 3.2 Statistical analysis

Design Expert software was used to create 27 runs for the experiments (Table 3). Four important variables catalyst loading, temperature, reaction time and methanol:oil ratio—were investigated as input parameters. ANOVA analysis (Table 4S) was performed to demonstrate the model's adequacy. The F-value and P-value are two very important parameters in the ANOVA analysis. In this way, the model P-value was less than 0.05, and the related F-value was high, indicating the adequacy of the model and the appropriateness of the polynomial quadratic equation [30,31]. The low value of C.V.% (2.15) and the values close to 1 for R^2^ (0.995), R^2^_adj_ (0.992), and R^2^_pre_ (0.983) are other reasons for the adequacy of the chosen model [30,32]. Also, the R^2^ value shows that 99 % of the changes have been evaluated by the model [31,33]. As a result, the values of R^2^, R^2^_adj_, and R^2^_pre_ are all near to one, confirming the existence of a correlation between actual and anticipated values [30,31,33].

**Table 3:** Experimental design and actual yield values.

Run	Temperature (°C)	Catalyst (wt%)	Methanol: oil (vol:vol)	Reaction Time (min)	Yield (%)
1	55	7	1.75	120	81.70
2	40	4	0.50	82.5	50.14
3	55	1	3.00	82.5	65.60
4	55	7	0.50	82.5	70.70
5	55	4	0.50	120	74.80
6	55	4	1.75	82.5	84.70
7	40	1	1.75	82.5	53.50
8	55	1	1.75	45	59.80
9	55	4	0.50	45	54.36
10	55	4	3.00	45	51.58 51.8
11	55	7	1.75	45	56.50
12	70	1	1.75	82.5	92.13
13	70	4	1.75	45	82.91
14	40	7	1.75	82.5	50.41
15	40	4	1.75	45	41.40
16	55	1	1.75	120	82.22
17	40	4	3.00	82.5	45.40
18	55	1	0.50	82.5	55.69
19	70	7	1.75	82.5	89.90
20	70	4	1.75	120	96.80
21	55	4	1.75	82.5	86.50
22	70	4	3.00	82.5	81.10
23	55	7	3.00	82.5	52.40
24	55	4	1.75	82.5	88.25
25	40	4	1.75	120	64.60 64.6
26	55	4	3.00	120	70.80
27	70	4	0.50	82.5	81.35

**Table 4:** Experimental design and actual yield values.

Run	Temperature (°C)	Catalyst (wt%)	Methanol: oil (vol:vol)	Reaction Time (min)	Yield (%)
1	55	7	1.75	120	81.70
2	40	4	0.5	82.5	50.14
3	55	1	3.00	82.5	65.60
4	55	7	0.50	82.5	70.70
5	55	4	0.50	120	74.80
6	55	4	1.75	82.5	84.70
7	40	1	1.75	82.5	53.50
8	55	1	1.75	45	59.80
9	55	4	0.50	45	54.36
10	55	4	3.00	45	51.58
11	55	7	1.75	45	56.50
12	70	1	1.75	82.5	92.13
13	70	4	1.75	45	82.91
14	40	7	1.75	82.5	50.41
15	40	4	1.75	45	41.40
16	55	1	1.75	120	82.22
17	40	4	3.00	82.5	45.40
18	55	1	0.50	82.5	55.69
19	70	7	1.75	82.5	89.90
20	70	4	1.75	120	96.80
21	55	4	1.75	82.5	86.50
22	70	4	3.00	82.5	81.10
23	55	7	3.00	82.5	52.40
24	55	4	1.75	82.5	88.25
25	40	4	1.75	120	64.60
26	55	4	3.00	120	70.80
27	70	4	0.50	82.5	81.35

According to the experimental design, the coded equation (Eq. (3)) for the quadratic model is equal to:

(3) $$1/\left(\text{Mass}\;\text{Yield}\;\text{of}\;\text{Biofuel}\right)=0.0116\;–\;0.0043A+0.0002B+0.0005C\;–\;0.0026D\;–\;0.0005\text{AC}+0.0017\text{AD}+0.0019\text{BC}+0.002A^{2}+0.0016B^{2}+0.0032C^{2}+0.0017D^{2}$$

**Fig. 10:**
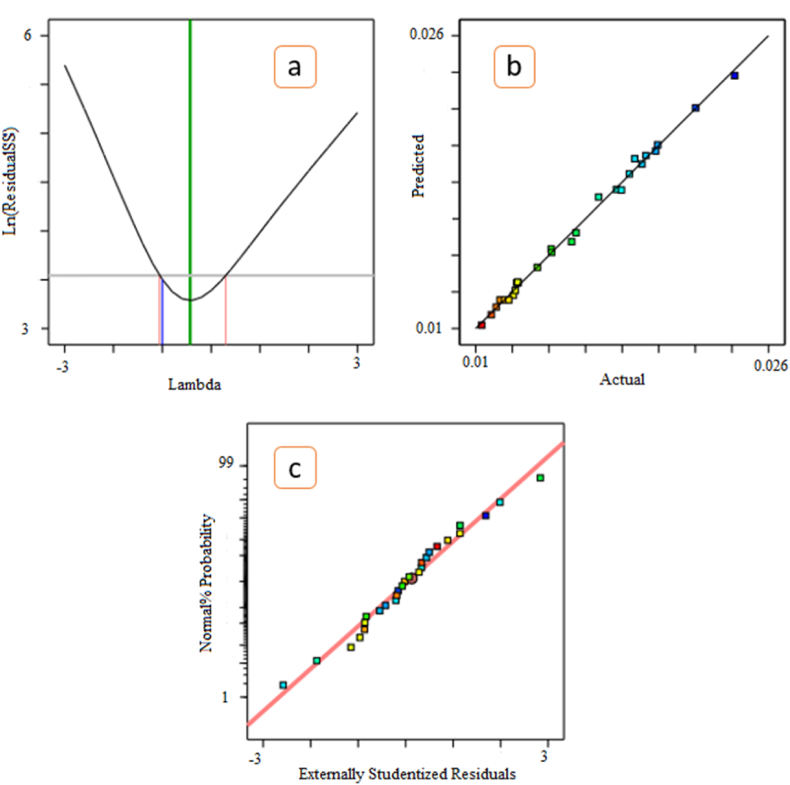
Transmission function (a), experimental values versus predicted values (b), and normal plot of residuals.

A positive value indicates a synergistic effect that favors optimization, while a negative sign represents an antagonistic effect or inverse effect of the factor on the selected response [34]. The best transfer function is determined through the Box-Cox plot (Fig. 10a). The best value of lambda is also at the lowest point of the Box-Cox plot, which is the least sum of squares that remains in the model [31]. As can be seen in Fig. 10b, there is a good correlation between the experimental and predicted values, and it has a good linear distribution. Fig. 10C also shows the normal distribution of the residuals, and an inappropriate linear distribution (S-like) indicates an inadequate model [31,35].

### 3.3 The influence of operational conditions on biodiesel production

**Fig. 11:**
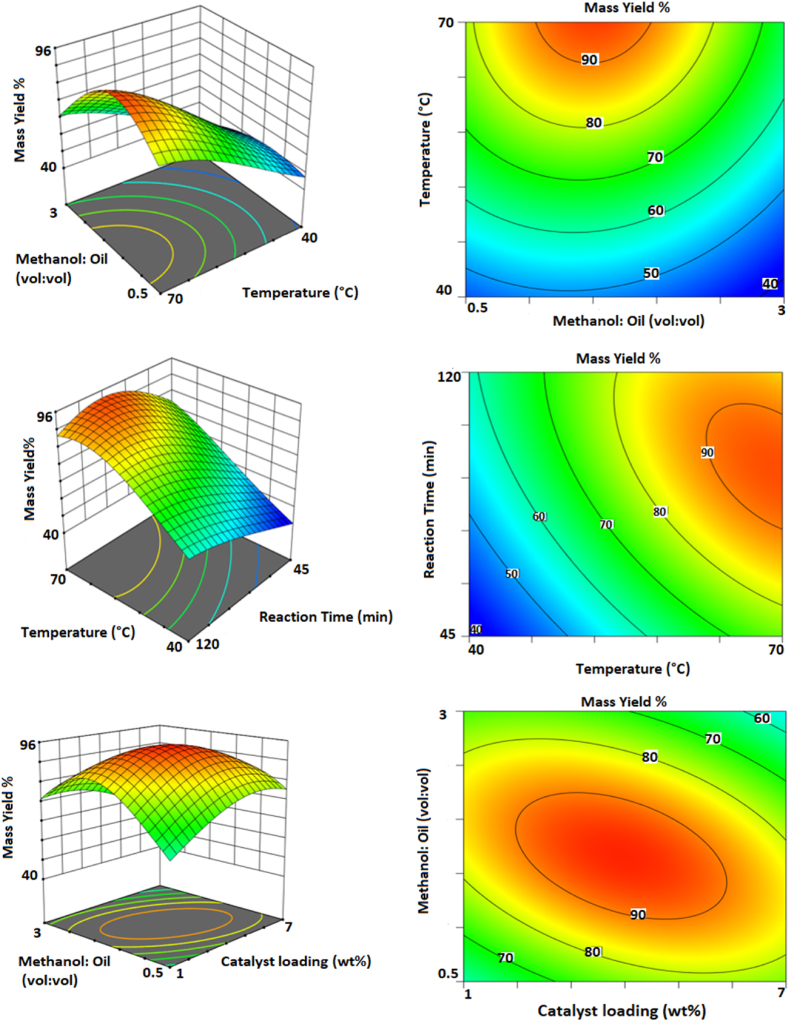
The mass yield of biofuel as a function of temperature, catalyst loading, and oil:MeOH ratio.

The impact of operational variables such as temperature, catalyst loading, methanol:oil ratio, and reaction time on the mass yield of biofuel production can be seen in Fig. 11. All parameters have a positive effect on the yield of the biofuel production process.

#### 3.3.1 Effect of catalyst on mass yield of biofuel

The reason for the rise in yield as the amount of catalyst increases is because the active sites of the catalyst increase [36]. Consequentially, more active sites participate in the transesterification process, enhancing biofuel output [37]. Also, the activation energy decreases (by increasing the active sites of the catalyst), which makes the reaction faster, and more free fatty acids (FFAs) are converted into fatty acid methyl esters (FAMEs) [11]. However, the excessive increase of the catalyst causes the miscibility of oil and methanol to decrease and the viscosity to increase, which ultimately leads to a decrease in biofuel production [18,20]. The reaction slurry viscosity is inversely proportional to the transfer rate of the reactants from the reaction medium to the catalyst surface [14]. Furthermore, using an insufficient quantity of catalyst (either too much or too little) results in the creation of soap, which decreases the mass yield of biofuel [11].

#### 3.3.2 Effect of methanol on mass yield of biofuel

The effect of the methanol: oil ratio is a very critical parameter that affects the efficiency of biodiesel production. A lower or higher ratio of methanol: oil has a significant effect on the conversion of triglycerides to methyl ester and may reduce the efficiency of biofuel production, and an inappropriate ratio of methanol leads to the formation of emulsions by glycerol and biodiesel formed during the reaction [38].Therefore, the reverse reaction and the recombination of glycerol and esters are created, and as a result, the efficiency of biodiesel decreases [38]. Due to its polar nature and short-chain structure, methanol is preferred over other alcohols in the transesterification process [7]. A very large amount of methanol used in transesterification reactions reduces the mass yield because it leads to the creation of a saponification process [7], a decrease in the concentration of the catalyst and reactants [15], and also changes the balance of the reaction. However, as can be seen in Fig. 11, with the increase in methanol, the yield also increases, which is because the reaction proceeds towards the creation of the product. However, the yield decreased with the addition of more methanol [36]. The pace of the reaction rises as the amount of methanol increases, causing regeneration of the active sites of the catalyst [39,40] as well as improved separation of the product from the catalyst's surface.

#### 3.3.3 Effect of temperature on mass yield of biofuel

Because the maximal reaction temperature should be near the boiling point of methanol, the reaction temperature range was set between 40 and 70 °C [23,26]. As the reaction temperature increases, the efficiency increases because the miscibility in the alcohol-oil phase increases and the reaction rate increases [23], leading to a rise in kinetic energy and the number of collisions between molecules [11], as well as improved mass transfer to the catalyst surface [3]. However, an excessive increase in the reaction temperature (above 110 °C) causes triglycerides to saponify, and bubbles are formed, which causes the reaction to fail to progress [41,42] and leads to reaction reversal [39]. Another reason for the negative effect of very high temperatures is that methanol evaporates and is removed from the reaction. For this reason, a suitable temperature (roughly, 65 °C) can be suitable for carrying out the transesterification reaction [40]. Also, transesterification reactions with methanol are usually carried out at temperatures close to the boiling point of alcohol [10].

#### 3.3.4 Effect of reaction time on mass yield of biofuel

Another critical component in the biofuel manufacturing process is reaction time. As seen in Fig. 11, the mass yield rises with increasing reaction time. The increase in mass yield is due to the existence of sufficient opportunity for the interaction of the reactants [43]. However, by further increasing the reaction time, a decrease in the yield of biofuel production can be observed. Because of the reversibility of the transesterification process, soap production is possible, and the amount of fatty acid methyl esters drops [11,42]. Furthermore, the process can be reversed by the hydrolysis of fatty acid methyl esters to free fatty acids [19,39].

### 3.4 Optimization

Based on the design and experiments, the input parameters were optimized. To achieve maximum efficiency in the processes, obtaining optimal values for the input factors is very important [30]. High F-values and low P-values for input parameters indicate the importance of these factors. For better optimization, the methanol:oil ratio and catalyst loading were selected in the range of 0.5–3 (vol:vol) and 1–7 (wt%). Temperature and reaction time parameters were set at their maximum values. Table 5 shows the optimum range for each parameter. Optimal circumstances for catalyst loading, temperature, reaction time, and methanol:oil ratio were obtained as 70 °C, 4.498 wt%, 1.968 (vol:vol), and 120 min, respectively. An experiment was conducted under optimal conditions, and the biofuel produced under these conditions was evaluated by GC-Mass analysis.

**Table 5:** Parameters range in numerical optimization.

Name	Goal	Lower Limit	Upper Limit
**Temperature (°C)**	Maximize	40	70
**Catalyst Loading (wt%)**	is in range	1	7
**Methanol: Oil (vol:vol)**	is in range	0.5	3
**Reaction time (min)**	Maximize	45	120
**Mass Yield of Biofuel (%)**	**Target = 96**	–	–

### 3.5 Biodiesel characterization

#### 3.5.1 Gas chromatography-mass spectrometry (GC-MS)

Table 6S illustrates the methyl esters produced by the MgO:CaO (70:30 wt%) calcined catalyst in the optimum conditions (temperature 70 °C, catalyst loading 4.498 wt%, methanol:oil ratio 1.968 (vol:vol), and time 120 min). There were 10 methyl esters found in the product, which were confirmed using the library match software. The biodiesel produced by the catalytic transesterification process has the greatest FAME content, 12-octadecenoic acid, ME, according to the GC-MS analysis.

#### 3.5.2 CHNS analysis, density, and viscosity

A biodiesel charactrization has a standard that explain if a biodiesel is applicable for using in a vehicle. Therefore, to prove that a biofuel is appropriate, its characterization must be compared with American Society for Testing and Materials (ASTM) standards. When the viscosity and density of biodiesel are outside the standard range, it can have a major impact on engine combustion. If the viscosity is too high, the fuel may not be atomized properly during injection. This means that fuel droplets are bigger and burn less efficiently, resulting in incomplete combustion. As a result, particle emissions may increase while engine performance decreases [44]. Biodiesel with low viscosity may produce over-atomization of the fuel, resulting in excessively small fuel droplets. This can lead to a faster burn rate and greater peak combustion temperatures. While this may improve combustion completeness, greater temperatures in the combustion chamber can lead to increased generation of nitrogen oxides (NO_x_) [44]. Higher density can influence the amount of fuel injected for a given volume, potentially resulting in an improper air-fuel ratio. This can lead to incomplete combustion, higher emissions, and worse engine efficiency. Higher viscosity in cold circumstances can potentially cause starting issues and greater wear on gasoline pumps and injectors [45]. Biodiesel with a low density has less mass per volume, which can affect the air-fuel ratio if the fuel injection system is not calibrated properly. This can result in a leaner mixture, thereby raising combustion temperatures and increasing NO_x_ emissions. Furthermore, decreasing energy content per unit volume can diminish engine power output [45]. One of the most important charachteristic of biodiesel is High Heating Value (HHV). Dulong's formula was used to determine HHV. The CHNS/O analysis was used to calculate HHV and simulate biodiesel combustion. The CHNS/O analysis measures the amount of oxygen, nitrogen, hydrogen, sulfur, and carbon in various materials. Different studies looked into the characteristics of biodiesels produced from various feeds like waste cooking oil [46]. Spirulina platensis algae biomass [47], and vegetable oils [48]. Table 7, Table 9 exhibit the findings of the CHNS study, biodiesel characteristics, and ASTM standards, respectively. Table 9 shows that the qualities of the produced biodiesel are within the ASTM standards range, similar to a study that measured and compared the properties of biodiesels made from coconut oil-based biodiesel, jatropha oil-based biodiesel, and waste oil-based biodiesel [49].

**Table 7:** Weight percent of each element in CHNS analysis (%w/w).

Element (%)	Value
**C**	72.668
**H**	11.228
**N**	5.882
**S**	0.001
**O**	10.221

### 3.6 External costs

The external costs associated with the production and use of fuels should be taken into account in addition to the production costs in order to determine the economic relevance and proper comparison of fossil fuels and biofuels. The environmental effects of burning diesel and biodiesel were examined using the Diesel-RK software. This software has been used to compare engine performance and emissions from the combustion of diesel and biodiesel. Fuels produced from algae [43]. The information that was used for the engine combustion modeling is in Table 8S [50]. The software library was used for diesel, and Table 9 was used for biodiesel.

**Table 9:** The amount of each parameter in this study and the ASTM standard.

Property	Biodiesel	ASTM standard
**Density at 15 °C (kg/m**^**3**^**)**	920	880
**Viscosity at 40 °C (cst)**	5.89	1.9–6
**C (%)**	77.21	77
**H (%)**	11.93	12
**O (%)**	10.86	11
**S (ppm)**	8	<500
**HHV**^a^**(MJ/kg)**	41.20	–

a Derived by Dulong's formula.

The environmental emissions from both fuels' simulation, the external costs resulting from each of the environmental emissions based on EPS2000 [50], and the prices for each fuel are listed in Table 10. The data in Table 10 demonstrate the superiority of biodiesel over diesel by showing that the total external costs due to environmental emissions for consumption of 1 L of diesel and biodiesel are 0.38887 € and 0.33499 €, respectively.

**Table 10:** Environmental pollution and external costs of diesel and biodiesel.

Pollutant	Environmental pollution from burning in engine (g/Lit _fuel_)		External costs (€/kg_emission_)	External costs of diesel and biodiesel (€/Lit _fuel_)	
	diesel	biodiesel	prices	diesel	biodiesel
**Carbon dioxide (CO**_**2**_**)**	2674.44444	2280.45688	0.108	0.28884	0.24639
**Particle matter (PM)**	0.66280	0.15815	36.100	0.02393	0.00571
**Nitrogen oxides (NO**_**x**_**)**	35.67744	38.72575	2.130	0.07599	0.08249
**Sulfur dioxide (SO**_**2**_**)**	0.03353	0.01276	3.300	0.00011	0.00004
**Total**	–	–	–	0.38887	0.33499

### 3.7 Recovery and stability of the catalyst

**Fig. 12:**
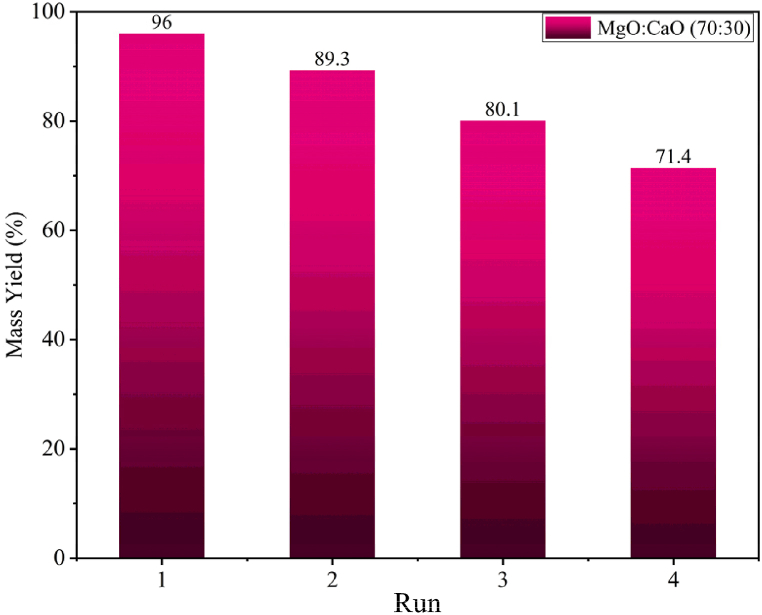
Reusability of magnesium/calcium oxide catalyst.

The catalyst's reusability was examined since it is a crucial and useful consideration in the process of choosing it [51]. The magnesium/calcium oxide catalyst was investigated in four consecutive cycles. After the first run, the catalyst was filtered, washed with methanol, and then placed in an oven at 120 °C for 4 h [36]. The catalyst was utilized for the second run once it had fully dried. Similarly, the catalyst was employed in a maximum of four investigations; Fig. 12 illustrates that the initial efficiency was 96 %, and following the fourth recovery and reuse, it dropped to roughly 71 %. The decrease in the efficiency and activity of the catalyst in intermittent runs has several reasons. We can list the leaching of the catalyst's active components in methanol as one of these causes, but the leaching of calcium and magnesium oxide in the form of Ca^2+^ and Mg^2+^ ions in short-chain alcohol is regarded as one of the less significant causes and will not significantly affect the catalyst's efficiency [52]. The catalyst's active sites becoming blocked as a result of poisoning in the reaction mixture is another factor that might cause efficiency to decrease [36,53,54].

Four transesterification tests were performed under the mentioned optimal conditions to check the active components of the catalyst. Then the three phases obtained were separated. Every time a sample was extracted from the liquid phase, it was subjected to the ICP-MS method for the examination of calcium and magnesium, as indicated in Table 11S [55].

### 3.8 Comparison of results with other research

In this section, the results of this study have been compared with other research. As can be seen in Table 12, other studies have used calcium oxide-based catalysts to convert various oils into biofuels. The catalysts used have the ability to produce biofuel from 73 % to 97 % yield. As it is known, the use of waste drugs as a heterogeneous catalyst in the production of biofuel has been able to show good performance and can be used as a suitable source in the preparation of heterogeneous catalysts in the future.

**Table 12:** Comparison of the effect of catalyst type in this study with other research.

No	Oil	Catalyst	Yield percentage	Reference
**1**	Sunflower	CaO	91 %	[56]
**2**	Waste Frying Oil	CaO	89 %	[57]
**3**	Cooking Oil	CaO-Based	73.53 %	[58]
**4**	Soybean Oil	CaO-Based	97.4 %	[34]
**5**	Waste Cooking Oil	MgO	95.6 %	[6]
		CaO	90.4 %	
**6**	Pistacia-Terebinthus (PT) Oil	MgO–CaO of Pharmaceutical Waste	96 %	This Study

## 4 Conclusion

In this study, calcination was utilized to provide a catalyst for the transesterification process using pharmaceutical wastes from calcium and magnesium tablets that are disposed in Iran through landfilling and incineration. The selection of these compounds has been done with the aim of trying to provide an alternative solution to the current waste management methods that cause harmful environmental issues such as soil and air pollution due to the burial and burning of this type of waste. PT oil, which has received less attention as a feed in this process, was used for the feed of the biofuel production process by transesterification. Methanol was also used as the alcohol required for this method to produce biofuel, which led to the production of fatty acid methyl esters with a mass efficiency of 96 % and a qualitative efficiency of 91.37 %. Catalyst loading, temperature, reaction time, and methanol:oil ratio are effective parameters that were investigated and optimized. Biofuel produced under optimal conditions was examined using GC-MS to determine the kinds and quantities of each of its methyl ester components. In order to quantify environmental emissions, Diesel-RK software performed a combustion simulation utilizing the density, viscosity, and CHNS/O analysis data. A comparison of the emissions produced by burning biodiesel and diesel fuel was also done by figuring out the external costs. As a result, the external costs of diesel and biodiesel were estimated at 0.38887 €/Lit and 0.33499 €/Lit, respectively, which shows that the external costs caused by the use of biodiesel are lower. The statistics of pharmaceutical waste amounts are only available for the year 2022 in Iran, and there are no separate statistics for them. For this reason, these factors can be considered limitations of this study. Therefore, these limitations make the use of pharmaceutical waste on a large scale challenging.

## Data availability

Data will be made available on request.

## CRediT authorship contribution statement

**Saman Rashidi:** Writing – original draft, Methodology, Investigation. **Ramin Tahmasebi-Boldaji:** Writing – original draft, Methodology, Investigation. **Aref Ahmadian Baghbadarani:** Writing – review & editing, Writing – original draft, Methodology, Investigation. **Majid Baghdadi:** Writing – review & editing, Methodology, Investigation. **Omid Tavakoli:** Methodology, Investigation. **Abdolreza Karbassi:** Project administration, Methodology, Investigation. **Akram Avami:** Investigation.

## Declaration of competing interest

The authors declare that they have no known competing financial interests or personal relationships that could have appeared to influence the work reported in this paper.
