# Supplementary material for: Biodiesel production through transesterification of waste Pistacia- Terebinthus Oil by pharmaceutical waste as a heterogeneous catalyst: A sustainable solution for reducing external costs
Source: Heliyon. 2024 Jul 17;10(14):e34404. doi: 10.1016/j.heliyon.2024.e34404 (PMC11325280; doi:10.1016/j.heliyon.2024.e34404)
Supplement: Multimedia component 1 [file mmc1.docx]

**Biodiesel Production through Transesterification of Waste Pistacia-
Terebinthus Oil by Pharmaceutical Waste as a Heterogeneous Catalyst: A Sustainable Solution for Reducing External Costs**

Saman Rashidi^a^ , Ramin Tahmasebi-Boldaji^b^ , Aref Ahmadian Baghbadarani^a^ , Majid Baghdadi^a^, Omid Tavakoli^c^, ^*^Abdolreza Karbassi^a^, Akram Avami^d^

^a^Department of Environmental Engineering, Graduate Faculty of Environment, University of Tehran, Tehran, Iran

^b^Department of Chemical Engineering, College of Engineering, University of Isfahan, P.O. Box 81746-73441, Isfahan, Iran

^c^School of Chemical Engineering, College of Engineering, University of Tehran, Tehran, 14176, Iran

^d^Department of Energy Engineering, Sharif University of Technology, Azadi Ave, Tehran, 14565-114, Iran

**^*^Corresponding author**: **Abdolreza Karbassi**

**Email address**: **[akarbasi@ut.ac.ir](mailto:akarbasi@ut.ac.ir)**

Department of Environmental Engineering, Graduate Faculty of Environment, University of Tehran, Tehran, Iran

**Authors:**

Saman Rashidi: [saman.rashidi@ut.ac.ir](mailto:saman.rashidi@ut.ac.ir)

Ramin Tahmasebi-Boldaji: [Ramintahmasbi68@gmail.com](mailto:Ramintahmasbi68@gmail.com)

Aref Ahmadian Baghbadarani: [aref.ahmadian.bag@ut.ac.ir](mailto:aref.ahmadian.bag@ut.ac.ir)

Majid Baghdadi: [m.baghdadi@ut.ac.ir](mailto:m.baghdadi@ut.ac.ir)

Abdolreza Karbassi: [akarbasi@ut.ac.ir](mailto:akarbasi@ut.ac.ir)

Omid Tavakoli: [otavakoli@ut.ac.ir](mailto:otavakoli@ut.ac.ir)

Akram Avami: [avami@sharif.edu](mailto:avami@sharif.edu)

**Table 4S.** ANOVA for the second-order polynomial model.

| Source | Sum of squares | df | F-value | P-value |
| --- | --- | --- | --- | --- |
| Model | 0.0004 | 11 | 324.3 | <0.0001 (**Significant)** |
| A- Temperature | 0.0002 | 1 | 2002.38 | <0.0001 |
| B-Catalyst loading | 5.995E-07 | 1 | 4.77 | 0.0452 |
| C- Methanol: Oil | 3.012E-06 | 1 | 27.67 | <0.0001 |
| D- Reaction Time | 0.0001 | 1 | 718.38 | <0.0001 |
| AC | 1.045E-06 | 1 | 9.60 | 0.0073 |
| AD | 0.0000 | 1 | 110.75 | <0.0001 |
| BC | 0.0000 | 1 | 134.5 | <0.0001 |
| A^2^ | 0.0000 | 1 | 204.35 | <0.0001 |
| B^2^ | 0.0000 | 1 | 128.79 | <0.0001 |
| C^2^ | 0.0001 | 1 | 495 | <0.0001 |
| D^2^ | 0.0000 | 1 | 134.52 | <0.0001 |
| Residual | 1.633E-06 | 15 | - | - |
| Lack of fit | 1.520E-06 | 13 | 2.07 | 0.3721  **(Not Significant)** |
| Pure Error | 1.128E-07 | 2 | - | - |
| Cor Total | 0.0004 | 26 | - | - |

**Table 6S.** GC-Mass analysis results for for biofuel.

| **Number** | **RT (min)** | **%Area** | **Hit Name** |
| --- | --- | --- | --- |
| **1** | 21.87 | 0.08 | Myristic acid, ME |
| **2** | 25.01 | 1.79 | Palmitoleic acid, ME |
| **3** | 25.65 | 20.21 | Palmitic acid, ME |
| **4** | 26.59 | 0.14 | Oleic acid, ME |
| **5** | 26.95 | 0.14 | Heptadecanoic acid, ME |
| **6** | 28.80 | 64.28 | 12-Octadecenoic acid, ME |
| **7** | 28.91 | 3.93 | Octadecanoic acid, ME |
| **8** | 31.08 | 0.20 | 11-Eicosenoic acid, ME |
| **9** | 34.04 | 0.15 | Docosanoic acid, ME |
| **10** | 31.41 | 0.45 | Eicosanoic acid, ME |

**Table 8S*.*** Information of the Mercedes OM642 3.0 diesel engine.

| **Items** | | **Information** | |
| --- | --- | --- | --- |
| **Cylinders** | 6 | |  |
| **Basic engine design** | V engine | |  |
| **Bore × Stroke** | 83mm × 92mm | |  |
| **Type of internal combustion engine** | Four_stroke cycle, turbocharged | |  |
| **Compression ratio** | 1 : 18 | |  |
| **Nominal engine speed** | 1500 rpm | |  |
|  | | | |

| **Run** | **Ca and Mg concentration in the solution (mgL^−1^)** | |
| --- | --- | --- |
|  | **Ca^2+^** | **Mg^2+^** |
| 1 | 4.52 | 7.44 |
| 2 | 4.68 | 7.86 |
| 3 | 6.22 | 8.24 |
| 4 | 6.86 | 9.58 |

**Table 11S.** Determination of the leached calcium and leached magnesium.
